# Supplementary material for: Significance of 5-S-Cysteinyldopa as a Marker for Melanoma
Source: Int J Mol Sci. 2020 Jan 9;21(2):432. doi: 10.3390/ijms21020432 (PMC7013534; doi:10.3390/ijms21020432)
Supplement: Supplementary file 1 [file ijms-21-00432-s001.pdf]

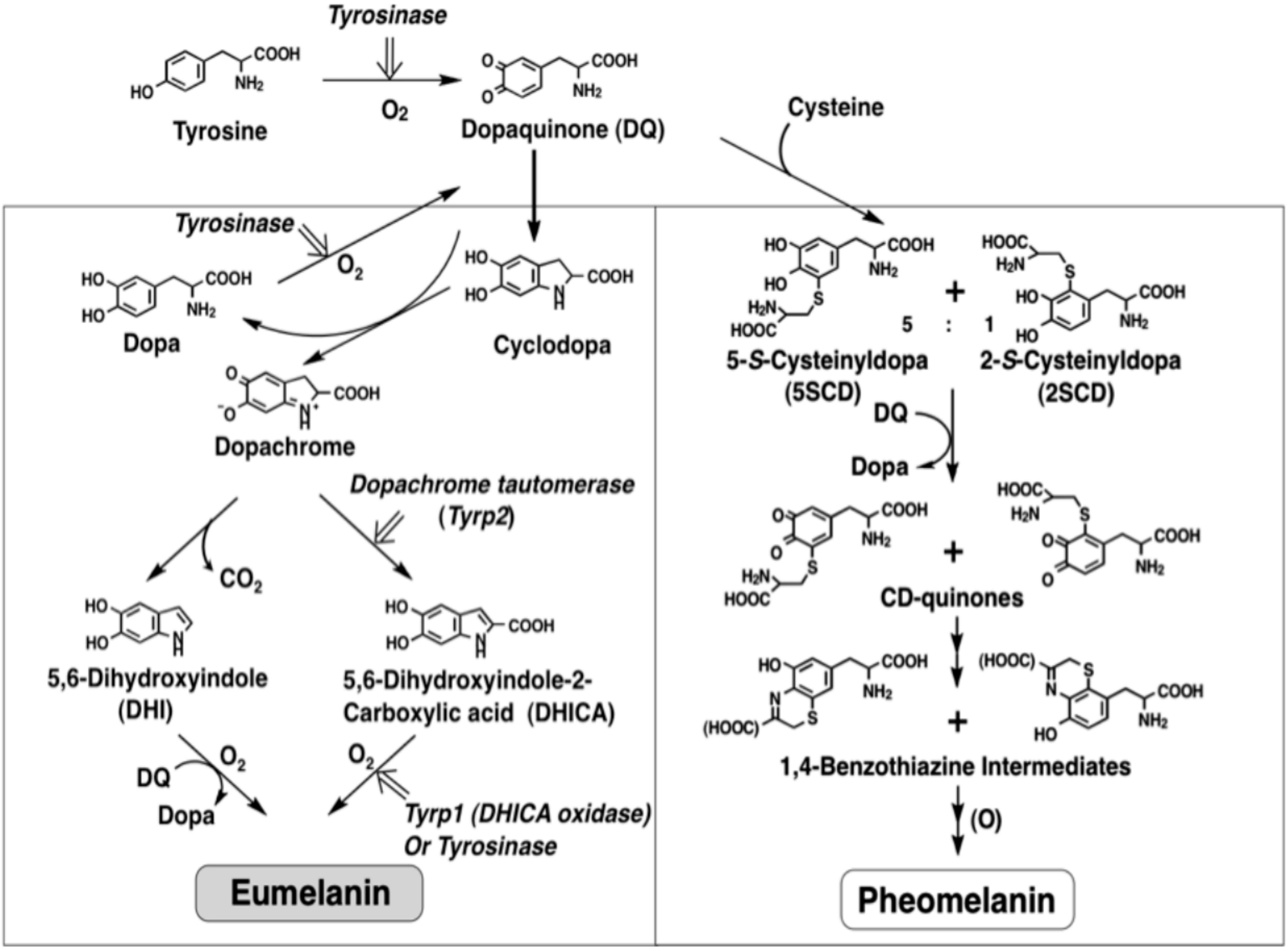


**Supplemental Figure S1. Biosynthesis pathway of eumelanin and pheomelanin.**

## Supplemental Table S1. Serum and urinary biomarkers for melanoma.

| Biomarker | Functions | References |
|---|---|---|
| VEGF (Vascular endothelial growth factor) | VEGF receptor ligand | 12 |
| FGF | FGF receptor ligand | 13 |
| EGF | EGF receptor ligand | 14 |
| Placental growth factor | Neuropilin-1 and -2 receptor ligand | 15 |
| TNF | G-protein-coupled receptor ligand | 16 |
| IL-8 | G-protein-coupled receptor ligand | 17 |
| Laminin-5 | Laminin receptor | 18 |
| Osteopontin | Integrin avb3 ligand | 19 |
| Tyrosinase | Essential enzyme in melanin synthesis | 20 |
| Gp100/pmel-17 | Melanin synthesis-associated melanosomal matrix glycoprotein | 21 |
| S100 proteins (S100B) | Cell division and differentiation-associated acidic calcium-binding protein | 22 |
| Serum amyloid A (SAA) | A superfamily of acute-phase proteins and proinflammatory adipokine | 23 |
| C reactive protein (CRP) | Tumor-associated inflammatory response | 24 |
| MIA | A small soluble protein | 25 |
| L-dopa/L-tyrosine | Precursors of melanin | 26 |
| TA-90 | Potential immunostimulatory or antineoplastic activator | 27 |
| Survivin | Apoptosis inhibition | 28 |
| Cytoplasmic/high-molecular-weight melanoma-associated antigen (CYT-MAA/HMW-MAA) | Melanoma progression | 29 |
| Galectin-3 | B-galactoside-binding protein | 30 |
| Gangliosides | Interactions between melanoma cells | 31 |
| Circulating-tumor DNA (ctDNA) | DNA methylation pattern | 32 |
| Micro-RNAs (miR) | Regulation of gene expression | 33 |
| Long non-coding RNAs | Regulation of gene expression | 34 |
| IL-1 | Survival or proliferation-associated factor | 35 |
| IL-4 | Survival or proliferation-associated factor | 36 |
| IL-6 | Survival or proliferation-associated factor | 37 |
| IL-10 | Survival or proliferation-associated factor | 38 |
| IL-12 | Survival or proliferation-associated factor | 39 |
| YKL-40 | Survival or proliferation-associated factor | 40 |
| Circulating melanoma cells (CMC) | Cancer cells released by tumor | 41 |
| Lactate dehydrogenase (LDH) | An indicator for liver metastasis | 42 |
| Glypican-3 | Survival or proliferation-associated factor | 43 |
| SPARC (Secreted protein acidic and rich in cysteine) | Matricellular protein | 44 |
| PKCa | Survival or proliferation-associated factor | 45 |
| 5SCD | Precursor of melanin | 46 |
| 6H5MI2C | Precursor of melanin | 47 |

**Supplemental Table S2. Patient characteristics**

| | | |
|---|---|---|
| Total number of patients | 218 | |
| Sex | | |
| Male | 112 | (51.4%) |
| Female | 106 | (48.6%) |
| Age[a] | | |
| Mean ± SD | 54.8 ± 15.3 years | |
| Range | 12-88 years | |
| Diagnosis | | |
| Acral lentiginous melanoma | 101 | (46.3%) |
| Nodular melanoma | 45 | (20.6%) |
| Superficial spreading melanoma | 43 | (19.7%) |
| Lentigo maligna melanoma | 11 | (5.0%) |
| Mucosal melanoma | 6 | (2.8%) |
| Uveal melanoma | 3 | (1.4%) |
| Unknown | 9 | (4.1%) |
| Site | | |
| Head and neck | 19 | (8.7%) |
| Trunk | 28 | (12.8%) |
| Leg | 30 | (13.8%) |
| Arm | 19 | (8.7%) |
| Foot | 103 | (47.2%) |
| Hand | 6 | (2.8%) |
| Mucous | 6 | (2.8%) |
| Ocular | 3 | (1.4%) |
| Unknown | 4 | (1.8%) |
| Stage | | |
| I | 25 | (11.5%) |
| II | 63 | (28.9%) |
| III | 112 | (51.4%) |
| IV | 18 | (8.3%) |
| Pigment production[b] | | |
| Melanotic | 158 | (72.5%) |
| Amelanotic | 12 | (5.5%) |
| Unknown | 48 | (22.0%) |

[a]At the initial visit.

[b]In primary lesion.

Adapted from Wakamatsu et al. (46)

**Supplemental Table S3. Frequency of elevated levels of serum 5SCD according to the presence or**

**absence of metastasis.**

| | No. | % |
|---|---|---|
| With distant metastasis (n = 62) | | |
| 5SCD all ≦ 10 nmol/L | 4 | 6 |
| 5SCD once > 10 nmol/L | 1 | 2 |
| 5SCD multiple > 10 nmol/L | 57 | 92 |
| With recurrence/local metastasis (n = 13) | | |
| 5SCD all ≦ 10 nmol/L | 8 | 62 |
| 5SCD once > 10 nmol/L | 4 | 31 |
| 5SCD multiple > 10 nmol/L | 1 | 7 |
| Without recurrence/metastasis (n = 143) | | |
| 5SCD all ≦ 10 nmol/L | 95 | 66 |
| 5SCD once > 10 nmol/L | 32 | 22 |
| 5SCD multiple > 10 nmol/L | 16 | 11 |

Adapted from Wakamatsu et al. (46)
